# Very late relapses in Hodgkin lymphoma treated with chemotherapy with or without radiotherapy: linear pattern and distinct prognostic factors

**DOI:** 10.1038/s41408-022-00674-w

**Published:** 2022-07-05

**Authors:** Theodoros P. Vassilakopoulos, Evrydiki Kravvariti, Fotios Panitsas, Maria K. Angelopoulou, Athanasios Liaskas, Flora N. Kontopidou, Xanthoula Yiakoumis, Eleni Variami, Maria N. Dimopoulou, Marina P. Siakantaris, John V. Asimakopoulos, Maria Arapaki, Maria Dimou, Panagiotis Diamantopoulos, Sotirios Sachanas, Chrysovalantou Chatzidimitriou, Marina Belia, Elianna Konstantinou, George Boutsikas, Kyriaki Petevi, Alexandros Kanellopoulos, Styliani Kokoris, Marie-Christine Kyrtsonis, Nora-Athina Viniou, Eleftheria Lakiotaki, Gerasimos Tsourouflis, Penelope Korkolopoulou, Kostas Konstantopoulos, Panayiotis Panayiotidis, Gerassimos A. Pangalis

**Affiliations:** 1 Department of Haematology and Bone Marrow Transplantation, National and Kapodistrian University of Athens, Laikon General Hospital, Athens, Greece; 2 First Department of Propaedeutic Internal Medicine, Joint Rheumatology Academic Program, National and Kapodistrian University of Athens, Laikon General Hospital, Athens, Greece; 3 Second Department of Internal Medicine, National and Kapodistrian University of Athens, Hippokratio General Hospital, Athens, Greece; 4 Department of Haematology, Athens Medical Center, Psychikon Branch, Athens, Greece; 5 First Department of Internal Medicine, National and Kapodistrian University of Athens, Laikon General Hospital, Athens, Greece; 6 First Department of Pathology, National and Kapodistrian University of Athens, Laikon General Hospital, Athens, Greece; 7 Second Propedeutic Department of Surgery, National and Kapodistrian University of Athens, Laikon General Hospital, Athens, Greece

**Keywords:** Hodgkin lymphoma, Risk factors

Hodgkin lymphoma (HL) is curable in most cases by modern chemotherapy with/without radiotherapy (CT ± RT). Most treatment failures represent either primary refractory disease or early relapses within 1 year (PR/ER). Relapses >1 year from treatment completion are considered “late relapses” (LR) associated with better outcomes [1]. The relapse rate gradually drops after the first year [2] and patients with sustained complete remission (CR) for >5 years are generally considered “cured”. However, relapses after >5 years (very late relapses (VLRs)) occasionally occur, and, until recently, VLRs after CT ± RT had been evaluated in rather small patient series [3–11]. In 2005, we initially analyzed the incidence of VLRs—those occurring >5 years from initial treatment initiation—and searched for relevant prognostic factors. In 2017, the German Hodgkin Study Group (GHSG) analyzed the incidence of VLRs in 4935 patients mainly treated with CT ± RT within the HD7-HD12 trials, reporting a linear pattern of continuous relapses up to 20 years [12]. However, it continuous to be unclear if this linear trend continues beyond 20 years without reaching a plateau and if baseline prognostic factors or treatment regimens or RT strategies affect the risk of VLR.

We attempted to shed light on these questions by analyzing our cohort of 1143 patients in sustained CR > 5 years after CT ± RT initiation, with >90% coming from a single center and treated mostly with ABVD or equivalent regimens (ABVDeq). The median follow-up was 13.8 years (IQR 9–20.7, range 5–47). The outcome of VLRs will be reported separately in a wider multicenter study [13].

Patients, treatment strategies and methods are described in detail in the Supplementary Material. The study flow-chart is shown in Supplementary Fig. 1. The primary endpoint was the cumulative incidence (CumInc) of VLR after the 60-month landmark from diagnosis considering the competing risk of death from any cause without prior relapse.

The baseline characteristics of the 1143 patients are shown in Supplementary Table 1. Among them 94% had cHL (nodular sclerosis (NS) 65%, mixed cellularity (MC) 24%), 91% received ABVDeq regimens and 73% received RT as combined modality treatment (CMT).

VLRs occurred in 66/1143 patients (2 as composite lymphoma). VLRs were initially compared to an additional group of 327 patients with PR/ER (*n* = 249) or LR 2–5 years (*n* = 78) (all defined from treatment initiation) (Supplementary Table 1). There was a monotonous drop of the frequencies of NS, ESR ≥ 50 mm/h (*p* < 0.001) and anemia (*p* = 0.008) from PR/ER to LR and VLR patients, while MC increased from 17 to 26% and 46% (*p* < 0.001). IPS was lower in VLRs compared to LRs and PR/ER (*p* = 0.004); advanced-stage, B-symptoms, leukocytosis, lymphocytopenia and hypoalbuminemia were overrepresented in PR/ER but did not differ between LR and VLR.

Among VLRs the median time-to-relapse was 9.3 years (IQR: 6.1–15, range 5–35). The distribution and CumInc of VLRs are presented in Supplementary Table 2 and Fig. 1A. The CumInc of VLRs showed continuous upward trend over time without signs of levelling off. The CumInc of VLR at 20 and 30 years from diagnosis for the whole patient population was 7.9% and 12.2% respectively. After treatment with ABVDeq ± RT it was 7.2 and 12.7% (Supplementary Table 3), and 7.2 and 13.6% for the 978 cHL patients (Supplementary Table 4 and Fig. 1E, F).

**Fig. 1:**
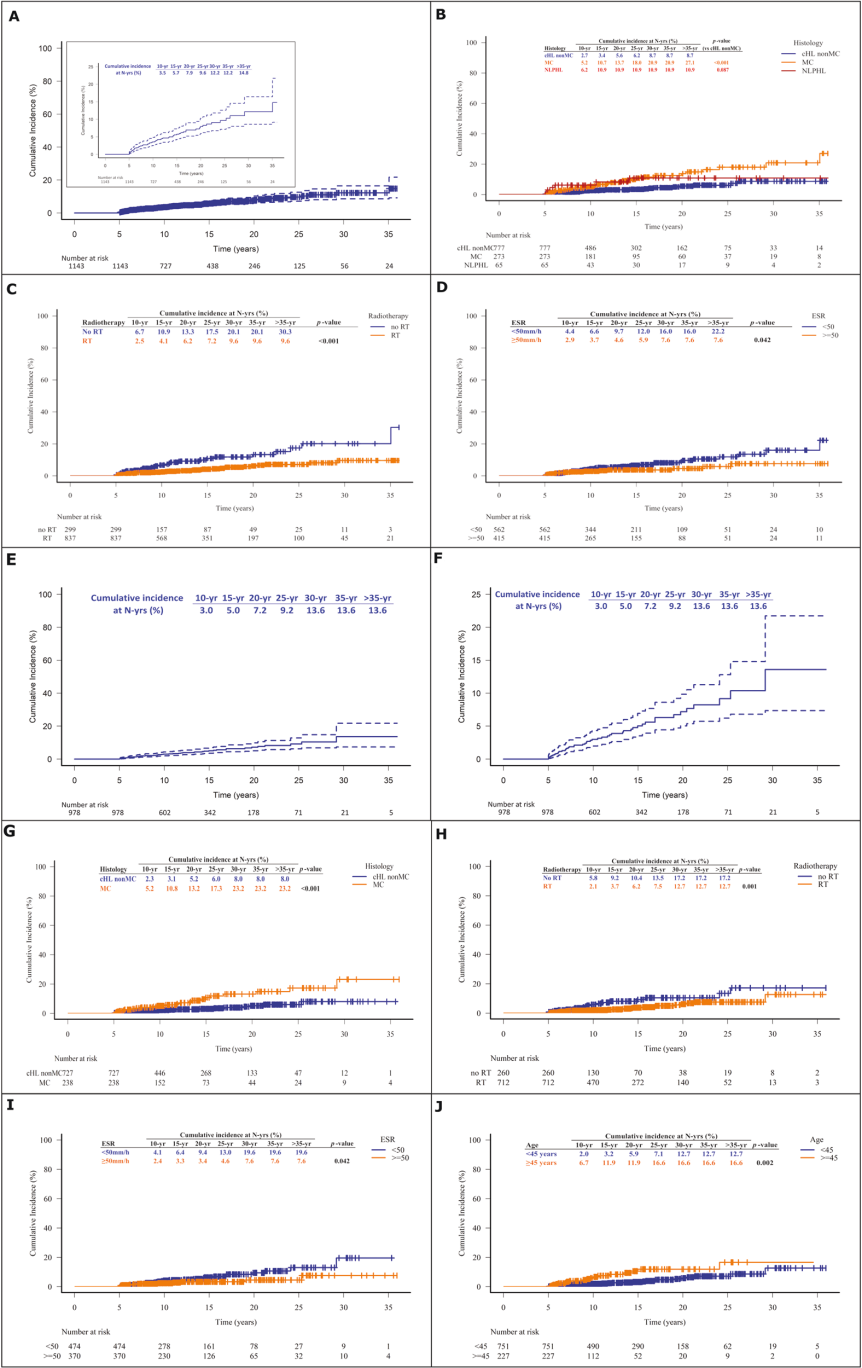
Cumulative incidence of very late relapses and competing-risks-adjusted cumulative incidence at different time-points from diagnosis in the whole patient population and in individual patients’ subgroups. Individual graphs refer to the whole patient population overall (**A**) and according to histologic subtype (cHL classical Hodgkin lymphoma, MC mixed cellularity, NLPHL nodular lymphocyte-predominant Hodgkin lymphoma) (**B**), radiotherapy (RT) administration (**C**) and erythrocyte sedimentation rate (ESR) (**D**). In addition, cumulative incidence of very late relapses and competing-risks-adjusted cumulative incidence at different time-points from diagnosis in patients with classical Hodgkin lymphoma treated with ABVD or equivalent regimens (**E**, **F**) and according to histologic subtype (cHL classical Hodgkin lymphoma, MC mixed cellularity) (**G**), radiotherapy (RT) administration (**H**), erythrocyte sedimentation rate (ESR) (**I**) and age (**J**).

Univariate and multivariate prognostic factor analysis is provided in Table 1. In univariate analysis of all patients, age ≥45 years and MC were associated with higher probability of VLRs, while ABVDeq vs. MOPPeq CT ± RT and CMT vs. CT alone were associated with lower VLR incidence (Table 1 and Supplementary Fig. 2 and Fig. 1C). Clinical stage and B-symptoms were of borderline significance (*p* = 0.11 and 0.13; Table 1); interestingly however, elevated ESR ≥ 50 mm/h was inversely associated with the incidence of VLR (Table 1 and Fig. 1D). Focusing on histology, MC was associated with higher VLR incidence compared to non-MC cHL, mainly driven by the favorable effect of NS, while NLP was marginally associated with more VLRs compared to non-MC cHL (Table 1 and Fig. 1B). In multivariate analysis (Table 1), independent protective factors for VLRs were CMT vs. CT (HR = 0.35, *p* < 0.001) and elevated ESR (≥50 vs. <50 mm/h; HR = 0.46, *p* = 0.008), while MC was independent adverse factor (HR = 2.19, *p* = 0.004). If ESR (with associated missing values) was not considered, ABVDeq CT vs. MOPP-type was protective against VLRs (HR = 0.50, 95% CI 0.27–0.94, *p* = 0.032) in the presence of other significant predictors (CMT/CT and MC) (data not shown).

**Table 1:** Univariate and multivariate competing-risks regression analysis of prognostic factors for very late relapse in the whole patient series, in ABVD treated patients and in ABVD treated patients with classical Hodgkin lymphoma.

	All patients (*N* = 1143, 66 events)				ABVDeq-treated patients (*N* = 1042, 50 events)				ABVDeq-treated patients with cHL (*N* = 978, 46 events)			
	Univariate analysis		Multivariate analysis^a^		Univariate analysis		Multivariate analysis^b^		Univariate analysis		Multivariate analysis^c^	
Variable	sHR (95% CI)	*p* value^d^	sHR (95% CI)	*p* value^d^	sHR (95% CI)	*p* value	sHR (95% CI)	*p* value^d^	sHR (95% CI)	*p* value	sHR (95% CI)	*p* value^d^
Age (≥45 vs. <45)	1.74 (1.04–2.90)	0.034	DNE		2.14 (1.20–3.81)	0.01	1.81 (0.93–3.51)	0.082	2.51 (1.39–4.53)	0.002	1.89 (0.95–3.74)	0.070
Gender (male vs. female)	1.43 (0.87–2.35)	0.155	DNE		1.44 (0.81–2.54)	0.211	DNE		1.81 (0.99–3.31)	0.053	DNE	
Stage (IB/IIB/III/IV vs. IA/IIA)	1.49 (0.92–2.43)	0.107	DNE		1.30 (0.74–2.30)	0.364	DNE		1.15 (0.64–2.09)	0.634	DNE	
B-symptoms (present vs. absent)	1.47 (0.90–2.41)	0.127	NE		1.35 (0.76–2.42)	0.306	NE		1.29 (0.70–2.36)	0.411	NE	
Histology												
(cHL vs. NLPHL)	0.67 (0.28–1.59)	0.359	NE		0.74 (0.25–2.14)	0.575	NE		NA		NA	
(MC vs. all other)	2.47 (1.52–4.00)	<0.001	2.19 (1.28–3.73)	0.004	2.67 (1.54–4.65)	0.001	2.20 (1.17–4.14)	0.015	NA		NA	
(MC vs. other cHL)	2.72 (1.64–4.52)	<0.001	NE		2.89 (1.63–5.15)	<0.001	NE		2.87 (1.61–5.12)	<0.001	2.21 (1.15–4.27)	0.018
(NLPHL vs. non-MC cHL)	2.21 (0.89–5.47)	0.087	NE		2.01 (0.67–6.05)	0.213	NE		NA		NA	
Chemotherapy (MOPPeq vs. ABVDeq)	0.49 (0.27–0.91)	0.024	DNE		NA		NA		NA		NA	
Radiotherapy (yes vs. no)	0.40 (0.24–0.64)	<0.001	0.35 (0.21–0.59)	<0.001	0.51 (0.29–0.89)	0.019	0.53 (0.28–1.03)	0.062	0.47 (0.26–0.84)	0.011	0.51 (0.26–1.01)	0.054
Anemia (present vs. absent)	1.11 (0.67–1.83)	0.686	NE		1.05 (0.59–1.86)	0.870	NE		0.93 (0.51–1.70)	0.811	NE	
WBC (≥ 10 vs. <10 × 10^9^/l)	0.69 (0.41–1.42)	0.147	NE		0.69 (0.39–1.24)	0.214	NE		0.55 (0.30–1.03)	0.061	DNE	
Lymphopenia (present vs. absent)	0.47 (0.12–1.89)	0.285	NE		0.59 (0.14–2.41)	0.462	NE		0.64 (0.16–2.60)	0.529	NE	
Albumin (<4 vs. ≥4 g/dl)	1.30 (0.73–2.30)	0.371	NE		1.19 (0.65–2.19)	0.574	NE		1.11 (0.58–2.11)	0.749	NE	
ESR (≥50 vs. <50 mm/h)	0.55 (0.31–0.99)	0.047	0.46 (0.26–0.82)	0.008	0.53 (0.27–1.04)	0.067	0.49 (0.25–0.96)	0.039	0.54 (0.27–1.06)	0.071	0.50 (0.25–0.997)	0.049
IPS (≥3 vs. <3)	1.08 (0.57–2.04)	0.815	NE		1.23 (0.63–2.42)	0.544	NE		1.31 (0.66–2.59)	0.445	NE	
VLR risk Score												
(1 vs. 0)	4.31 (1.00–18.56)	0.050	NA		2.94 (0.67–13.00)	0.155	NA		2.68 (0.59–12.05)	0.200	NA	
(2 vs. 0)	7.84 (1.82–33.67)	0.006	NA		6.56 (1.52–28.35)	0.012	NA		6.75 (1.56–29.23)	0.011	NA	
(3–4 vs. 0)	15.35 (3.54–66.58)	<0.001	NA		11.47 (2.55–51.64)	0.001	NA		11.53 (2.56–52.00)	0.001	NA	

*sHR* sub hazard ratio, *95% CI* 95% confidence intervals, *cHL* classical Hodgkin lymphoma, *DNE* did not enter the model, *NE* not evaluated, *NA* not applicable, *NS* nodular sclerosis, *MC* mixed cellularity, *NLPHL* nodular lymphocyte-predominant Hodgkin lymphoma, *MOPPeq* MOPP or equivalent regimen, *ABVDeq* ABVD or equivalent regimen, *ESR* erythrocyte sedimentation rate, *IPS* International Prognostic Score, *VLR* very late relapses, *VLR risk score* no of risk 4 factors (MC histology, ESR < 50 mm/h, no RT, age ≥ 45 years).

Also evaluated in the model:

^a^Age, gender, stage, treatment with ABVD or equivalents vs. MOPP or equivalents.

^b^Gender, stage.

^c^Gender, stage, leukocytosis.

^d^Fine and Gray model.

If the analysis was restricted to patients treated with anthracycline-based CT (*n* = 1042 including 50 VLRs), both MC (HR = 2.20, *p* = 0.015) and age ≥45 years (HR = 1.81, *p* = 0.082) entered the final backward model as independent adverse prognostic factors, while elevated ESR (≥50 vs. <50 mm/h; HR = 0.49, *p* = 0.039) and CT + RT vs. CT (HR = 0.53, *p* = 0.062) were selected as independent protective factors in multivariate analysis (Table 1).

In multivariate analysis focusing to patients with cHL treated with anthracycline-based CT (*n* = 978 including 46 VLRs), both MC (HR = 2.84, *p* = 0.001) and age ≥45 years (HR = 1.99, *p* = 0.031) were independent prognostic factors (Table 1). Finally, when the analysis was restricted to patients treated after 1996, the significance of MC was confirmed (HR = 2.91, *p* = 0.009) and use of RT was retained in the backward model as a protective factor (HR = 0.45, *p* = 0.058) (data not shown).

Since all estimated HR of the four independent predictors in the final backward multivariate model in ABVDeq-treated subgroup were roughly similar (~2.00 or 0.50), we assigned one point to each unfavorable factor, namely MC, age ≥45 years, ESR < 50 mm/h and omission of RT. The resulting score separated the three cohorts (all patients, ABVDeq-treated and cHL ABVDeq-treated) into four adequate-sized subgroups (0, 1, 2, 3–4 factors) with significantly divergent outcomes (Table 1 and Supplementary Tables 2–4 and Supplementary Fig. 3a–c).

VLRs of HL have been long recognized since 1985 [3] and recorded as late as 30–32 years after diagnosis with 2/3 such cases being NLPHL [14, 15]. Three VLR studies in the 90 s included patients with variable definition (>2, 4 or 5 years), mainly treated with RT alone and/or outdated chemotherapy [4–6]. RT alone was a risk factor for VLR but these results are not applicable in the modern treatment era.

In the era of modern CT ± RT, four small-to-medium sized published studies have roughly included 300–450 patients and reported 16–30 VLRs each, with the most delayed ones recorded at 16.5–22 years [7–10]. Thus they may have not been adequately powered to detect relevant prognostic factors.

In 2017, the GHSG reported a linear VLR risk for 20 years, which was lowest in the advanced-stage trials, suggesting that BEACOPP-based strategies not only reduce PR/ER and LRs but also VLRs, while VLRs were highest in early-stage trials delivering the least intensive approaches, in line with other reports in the 90s [4, 6, 10]. Only demographics, risk classification and trial generation were evaluated and increasing age, male gender and early stages were independent predictors of VLR.

The present study provided novel observations in an attempt to shed light on some of the questions raised above, which remained unanswered even after the large GHSG study.

First, we demonstrated that the linear pattern of VLRs continues beyond 20 years up to 25–30 years. Interestingly, we recorded the latest VLR at 35.1 years and recorded 8 cases beyond the 20th year.

Second, patients who did not receive RT were more likely to experience VLRs. If this is validated, it may impact the long-term results of recent randomized trials, which are awaited with great interest, because non-inferiority of CT vs. CMT may be affected.

Third, elevated ESR ≥ 50 mm/h was associated with lower VLR risk despite the marginally positive association of its strong correlates (stage and B-symptoms) with VLRs (HR ~1.2–1.5). Elevated ESR denotes biologically active, more aggressive disease, which may have a different biology related to PR/ER but also with some kind of long-term plateau and lower risk of VLR. Thus, some patients with advanced disease, probably those with delayed diagnosis and less inflammatory background reflected by lower ESR, may be more prone to the development of VLR. This hypothesis is supported by the progressively decreasing percentage of ESR ≥ 50 mm/h in patients with PR/ER, LR and VLR (Supplementary Table 1).

Lastly, our most striking finding was that the MC subtype was by far the most likely to be complicated by VLRs in sharp contrast with NS, with additional increasing MC and decreasing NS frequencies across the PR/ER, LR and VLR categories (Supplementary Table 1). To confirm this strong association, we repeated the analysis in the subgroup of patients diagnosed after 1996, when the REAL and WHO classifications diagnostic criteria were implemented in the pathology labs in Greece. Notably, the potential association between histology and VLRs had not been statistically evaluated in any of the other reported studies. Whether Epstein-Barr virus, overrepresented in MC, is involved in the association with VLRs deserves further investigation.

In conclusion, the present study provides new information by revealing the linear pattern of continuing relapses for up to 25–30 years, describing cases with extremely late relapses up to 35 years, confirming these findings in patients specifically treated with ABVDeq and, most importantly, uncovering previously unrecognized prognostic factors for VLRs, which may not only impact the follow-up strategies and patient counseling but also highlight the unique biology of different histologic subtypes, especially MC. Obviously, this information may be modified with PET-driven strategies and the incorporation of novel agents into the first-line therapy.

## Supplementary information

Supplementary Material
