# Supplementary material for: Very late relapses in Hodgkin lymphoma treated with chemotherapy with or without radiotherapy: linear pattern and distinct prognostic factors
Source: Blood Cancer J. 2022 Jul 5;12(7):102. doi: 10.1038/s41408-022-00674-w (PMC9256621; doi:10.1038/s41408-022-00674-w)
Supplement: Supplementary file 1 — Supplementary Material [file 41408_2022_674_MOESM1_ESM.docx]

**Supplementary Material**

[**Patients and Methods** 2](#_Toc97373597)

[**Supplementary Table 1** 4](#_Toc97373598)

[**Supplementary Table 2** 5](#_Toc97373599)

[**Supplementary Table 3** 7](#_Toc97373600)

[**Supplementary Table 4** 9](#_Toc97373601)

[**Supplementary Figure 1** 11](#_Toc97373602)

[**Supplementary Figure 2** 12](#_Toc97373603)

[**Supplementary Figure 3** 14](#_Toc97373604)

[**References** 15](#_Toc97373606)

# **Patients and Methods**

**Patients**

Among 1637 consecutive patients with HL treated between 1976 and early 2016, 23 received RT alone and 1614 received CT±RT at the Department of Haematology and other Departments of the Laikon General Hospital, National and Kapodistrian University of Athens (NKUA; n=1463), the Department of Haematology - Athens Medical Center (n=133), and the 2^nd^ Department of Internal Medicine of the NKUA, “Hippookration” Hospital (n=18). Among them, 1143 were alive and disease-free at 5 years following the initiation of CT and were eligible for analysis.

**Treatment Strategies**

Treatment strategies for early and advanced HL have been described previously^1-5^. Early-stage patients were offered combined modality therapy including low-dose involved-field RT. Initially they were treated with the MOPP regimen^6^ (nitrogen mustard, vincristine, procarbazine, prednisone), but a minority received MOPP-equivalents, such as ChlVPP (chlorambucil, vinblastine, procarbazine, prednisone) or COPP (cyclophosphamide instead of nitrogen mustard). Between 1988 and 1991, early-stage patients received EBVD^7^ [epirubicin 40mg/m^2^ instead of adriamycin 25mg/m^2^, bleomycin, vinblastine and dacarbazine 300-400 mg iv, considered as ABVD-equivalent. Roughly after 1998 all patients were treated with ABVD. RT was routinely administered to early-stage patients being omitted in a minority based on patient’s preference or medical contraindications.

All advanced-stage patients received MOPP or equivalents until 1980. Thereafter, from 1981 until 1997, advanced-stage patients were successively treated with alternating MOPP/ABVDx12, MOPP/ABV or MOPP/EBV hybridx6, alternating MOPP/ABVDx6 or even EBVD; all these regimens were considered as ABVD-equivalents^8,9^. Beyond 1998, ABVD became the standard regimen. After late 2008, most advanced-stage patients treated in NKUA Departments received ABVDx2 followed by interim positron emission tomography (PET)-based treatment modification to BEACOPP-escalated^10^. Overall, 16 deviations from ABVD during the period 1998-2016 included 9 patients who received BEACOPP-escalatedx2 followed by ABVDx6 prior to the introduction of PET and 7 additional patients who received BEACOPP-escalatedx4-6 after a positive interim PET. Adjuvant low-dose involved-field radiotherapy was administered to a significant proportion of stage IIB patients and a minority of stage III patients. In addition, sites of residual disease were irradiated with standard doses (~4000cGy), irrespectively of clinical stage.Thus RT was administered to ~60% of patients with AAS IB, IIB and IIIA but only a minority of 30% of AAS IIIB/IV, usually to sites of residual disease.

**Statistical Analysis**

Median values, range and interquartile range (IQR) were used to summarize continuous variables and frequencies and percentages for categorical variables. Comparisons between categorical variables were performed with Pearson’s chi-square test. Median follow-up was estimated using the reverse Kaplan-Meier method^11^.

The primary endpoint was the cumulative incidence (CumInc) of VLR after the landmark time of 60 months from diagnosis, considering the competing risk of death from any cause without prior relapse. Cases alive in continuous first remission were censored at last follow-up. CumInc curves were estimated nonparametrically and compared using the Gray test^12^. Regression modelling was done with the subdistribution relative hazard method of Fine and Gray^13^. The variables shown in tables 1 and 2 were analyzed at the reported cutoffs. Predictors showing univariate significance at the 0.10 level were included in the initial (full) multivariate model, but age, gender, stage, chemotherapy regimen, RT and histologic subtype were included in the full model, irrespective of univariate significance, as a measure to control for potential confounding. Only cases with complete data for all variables considered in the initial model were used in final model selection. The final model was reached by stepwise backward selection, setting entry probability at 0.05 and removal probability at 0.10.

Since all estimated hazard ratios (HR) of the independent predictors in the final model in the ABVDeq-treated subgroup were roughly similar, we assigned a score value ranging from 0 to 4, according to the total number of these adverse characteristics, to each case. Cases with scores 3 and 4 were merged. The new categorical variable created had four levels (0, 1, 2, 3-4) and was tested in a Fine and Gray model. The discriminating power of the score was assessed with the concordance index (Harrell’s c index)^14^. To obtain more reliable confidence intervals of the index, we randomly split the sample into a training set and a test set and estimated the Harrell’s c index in the test set after fitting the model to the training set^15^. Proportionality assumption was assessed using analysis of Shoenfeld residuals^16^.

Statistical analysis was performed with STATA version 11.2 (StataCorp. 2009. Stata Statistical Software: Release 11. College Station, TX: StataCorp LP) and EZR v 1.54^17^

# **Supplementary Table 1**

Baseline demographic, clinical, laboratory and treatment characteristics of 1143 patients in continuous CR1 for >5 years and comparison among very late relapses, late relapses and early relapses/primary refractory.

| **Baseline Characteristics** | | **All Patients**  **(N=1143)** | | **Very Late Relapses  (>5 yrs; N=66)** | | ***p-*value*** | **Late Relapses (2-5 yrs; N=78)** | | **Early Relapses/Prog-ression (<2yrs; N=249)** | | ***p-*value**** |
| --- | --- | --- | --- | --- | --- | --- | --- | --- | --- | --- | --- |
|  | | **#** | **%** | **#** | **%** |  | **#** | **%** | **#** | **%** |  |
| Age | <45 yrs | 867 | 76 | 44 | 67 | 0.072 | 54 | 69 | 177 | 71 | 0.775 |
|  | ≥45 yrs | 276 | 24 | 22 | 33 |  | 24 | 31 | 72 | 29 |  |
| Gender | Male | 622 | 54 | 41 | 62 | 0.205 | 47 | 60 | 140 | 56 | 0.623 |
|  | Female | 521 | 46 | 25 | 38 |  | 31 | 40 | 109 | 44 |  |
| Stage | IΑ/IIA | 679 | 60 | 32 | 49 | 0.070 | 36 | 46 | 70 | 28 | 0.001 |
|  | ΙΒ/IIB/III/IV | 462 | 40 | 34 | 51 |  | 42 | 54 | 179 | 72 |  |
| B-symptoms | No | 791 | 69 | 39 | 59 | 0.063 | 51 | 65 | 112 | 45 | 0.003 |
|  | Yes | 350 | 31 | 27 | 41 |  | 27 | 35 | 137 | 55 |  |
| Histology | NLPHL | 65 | 6 | 6 | 9 | 0.217 (NLP vs cHL)  0.001 (overall) | 6 | 8 | 11 | 5 | 0.269 (NLP vs cHL)  <0.001 (overall) |
|  | All cHL | 1062 | 94 | 59 | 91 |  | 70 | 92 | 232 | 95 |  |
|  | NS | 722 | 65 | 26 | 40 |  | 43 | 57 | 180 | 74 |  |
|  | MC | 273 | 24 | 30 | 46 |  | 20 | 26 | 40 | 17 |  |
|  | LD | 8 | 1 | 0 | 0 |  | 1 | 1 | 6 | 3 |  |
|  | LR | 37 | 3 | 3 | 5 |  | 4 | 5 | 0 | 0 |  |
|  | Unclassifiable | 13 | 1 | 0 | 0 |  | 2 | 3 | 5 | 2 |  |
| Chemotherapy | MOPP-eq | 101 | 9 | 16 | 24 | <0.001 | 11 | 14 | 34 | 14 | 0.100 |
|  | ABVD-eq | 1042 | 91 | 50 | 76 |  | 67 | 86 | 215 | 86 |  |
| Radiotherapy | No | 299 | 26 | 29 | 44 | 0.001 | 32 | 44 | 91 | 54 | 0.181 |
|  | Yes | 837 | 74 | 37 | 56 |  | 41 | 56 | 76 | 46 |  |
| Anemia | No | 714 | 64 | 41 | 63 | 0.923 | 44 | 56 | 108 | 44 | 0.008 |
|  | Yes | 408 | 36 | 24 | 37 |  | 34 | 44 | 139 | 56 |  |
| Leukocyte count | <10x10^9^/L | 653 | 59 | 43 | 66 | 0.195 | 49 | 63 | 124 | 50 | 0.024 |
|  | ≥10x10^9^/L | 464 | 41 | 22 | 34 |  | 29 | 37 | 123 | 50 |  |
| Lymphopenia | No | 952 | 92 | 57 | 97 | 0.172 | 69 | 93 | 192 | 82 | 0.002 |
|  | Yes | 84 | 8 | 2 | 3 |  | 5 | 7 | 43 | 18 |  |
| Albumin | ≥4 g/dL | 504 | 55 | 23 | 50 | 0.488 | 31 | 48 | 67 | 33 | 0.022 |
|  | <4 g/dL | 413 | 45 | 23 | 50 |  | 33 | 52 | 135 | 67 |  |
| ESR | <50mm/h | 562 | 57 | 38 | 70 | 0.049 | 30 | 46 | 72 | 34 | <0.001 |
|  | ≥50mm/h | 415 | 43 | 16 | 30 |  | 35 | 54 | 141 | 66 |  |
| IPS | <3 | 792 | 78 | 45 | 79 | 0.863 | 45 | 62 | 124 | 55 | 0.004 |
|  | ≥3 | 223 | 22 | 12 | 21 |  | 28 | 38 | 101 | 45 |  |

*NLPHL =Nodular Lymphocyte-Predominant Hodgkin Lymphoma, NS= Nodular Sclerosis, MC= Mixed Cellularity, LD= Lymphocyte Depleted, LR= Lymphocyte Rich, MOPP-eq= MOPP equivalent, ABVD-eq: ABVD equivalent, ESR= Erythrocyte Sedimentation Rate, IPS= International Prognostic Score*

**Comparison between 66 patients with VLRs and 1077 patients in continuous CR1 for at least 5 years*

***Comparison among 66 patients with VLRs, 78 patients with late relapses (2-5 years) and 249 patients with early relapse/progression (<2 years)*

# **Supplementary Table 2**

Rates of very late relapse at different time-points and univariate prognostic factor competing risks regression analysis for the whole patient series (n=1143).

| **Overall outcomes** | **10-year** | **15-year** | **20-year** | **25-year** | **30-year** | **35-year** | **40-year** | **Overall** | |  |
| --- | --- | --- | --- | --- | --- | --- | --- | --- | --- | --- |
| Pts remaining at-risk  at each time point | 731 | 442 | 248 | 140 | 58 | 27 | 7 | 1143 | |  |
| Cumulative number of VLRs until each time point | 36 | 50 | 58 | 62 | 65 | 65 | 66 | 66 | |  |
| Cumulative incidence  (95% CI) | 3.6%  (2.5-4.8 | 5.8%  (4.3-7.5) | 7.9%  (5.9-10.3) | 9.6%  (7.1-12.6) | 12.2%  (8.6-16.4) | 12.2%  (8.6-16.4) | 14.8%  (9.2-21.7) | 14.8  (9.2-21.7) | |  |
|  | | | | | | | | | | |
| **Cumulative incidence according to patients’ characteristics (%)** | | | | | | | | | **sHR (95% CI)** | ***p*- value*** |
| **Age** | | | | | | | | | | |
| <45 years (ref cat) | 2.8 | 4.5 | 6.9 | 7.8 | 11.1 | 11.1 | 14.3 | 1.74  (1.04-2.90) | | 0.034 |
| ≥45 years | 6.2 | 9.9 | 11.5 | 16.2 | 16.2 | 16.2 | 16.2 |  |  |  |
| **Gender** | | | | | | | | | | |
| Male | 4.4 | 6.5 | 9.6 | 12.1 | 14.2 | 14.2 | 14.2 | 1.43  (0.87-2.35) | | 0.155 |
| Female(ref cat) | 2.5 | 4.9 | 6.1 | 6.9 | 9.8 | 9.8 | 15.8 |  |  |  |
| **Stage** | | | | | | | | | | |
| I/IIA(ref cat) | 2.8 | 4.3 | 7.1 | 8.3 | 12.7 | 12.7 | 12.7 | 1.49  (0.92-2.43) | | 0.107 |
| IIB/III/IV | 4.6 | 8.0 | 9.3 | 11.5 | 12.7 | 12.7 | 16.6 |  |  |  |
| **B-symptoms** | | | | | | | | | | |
| No(ref cat) | 2.9 | 4.5 | 7.8 | 8.9 | 12.3 | 12.3 | 12.3 | 1.47  (0.90-2.41) | | 0.127 |
| Yes | 5.1 | 8.5 | 8.5 | 11.3 | 12.8 | 12.8 | 18.0 |  |  |  |
| **Histology** | | | | | | | | | | |
| NS | 2.6 | 3.0 | 5.2 | 5.9 | 8.5 | 8.5 | 8.5 |  | | |
|  | | | | | | | | | | |
| MC | 5.2 | 10.7 | 13.7 | 18.0 | 20.9 | 20.9 | 27.1 | 2.72  (1.64-4.52) | | <0.001 |
| Non MC cHL(ref cat) | 2.7 | 3.4 | 5.6 | 6.2 | 8.7 | 8.7 | 8.7 |  |  |  |
|  | | | | | | | | | | |
| NLPHL | 6.2 | 10.9 | 10.9 | 10.9 | 10.9 | 10.9 | 10.9 | 2.21  (0.89-5.47) | | 0.087 |
| Non MC cHL(ref cat) | 2.7 | 3.4 | 5.6 | 6.2 | 8.7 | 8.7 | 8.7 |  |  |  |
| **Chemotherapy** |  | | | | | | | | | |
| MOPP-eq(ref cat) | 8.0 | 11.2 | 14 | 15.6 | 17.2 | 17.2 | 20.7 | 0.49  (0.27-0.91) | | 0.024 |
| ABVD-eq | 3.1 | 5.2 | 7.2 | 8.9 | 12.7 | 12.7 | 12.7 |  |  |  |
| **Radiotherapy** | | | | | | | | | | |
| No(ref cat) | 6.7 | 10.9 | 13.3 | 17.5 | 20.1 | 20.1 | 30.3 | 0.40  (0.24-0.64) | | <0.001 |
| Yes | 2.5 | 4.1 | 6.2 | 7.2 | 9.6 | 9.6 | 9.6 |  |  |  |
| **Anemia** |  | | | | | | | | | |
| No(ref cat) | 3.3 | 4.6 | 7.7 | 9.7 | 13.3 | 13.3 | 16.3 | 1.11  (0.67-1.83) | | 0.686 |
| Yes | 4.0 | 7.8 | 8.5 | 9.7 | 9.9 | 9.9 | 9.9 |  |  |  |
| **Leukocyte count** | | | | | | | | | | |
| <10x10^9^/L(ref cat) | 4.4 | 6.5 | 9.0 | 11.0 | 12.5 | 12.5 | 16.7 | 0.69  (0.41-1.42) | | 0.147 |
| ≥10x10^9^/L | 2.3 | 4.7 | 6.8 | 8.1 | 12.2 | 12.2 | 12.2 |  |  |  |
| **Lymphopenia** | | | | | | | | | | |
| No(ref cat) | 3.6 | 6.1 | 8.5 | 9.5 | 12.4 | 12.4 | 15.3 | 0.47  (0.12-1.89) | | 0.285 |
| Yes | 2.2 | 2.2 | 2.2 | 16.0 | 16.0 | 16.0 | 16.0 |  |  |  |
| **Albumin** |  | | | | | | | | | |
| ≥4 g/dL(ref cat) | 3.2 | 4.8 | 7.6 | 8.8 | 8.8 | 8.8 | 8.8 | 1.3  (0.73-2.30) | | 0.371 |
| <4 g/dL | 3.6 | 6.2 | 7 | 10.4 | 12.9 | 12.9 | 21.2 |  |  |  |
| **ESR** | | | | | | | | | | |
| <50mm/h(ref cat) | 4.4 | 6.6 | 9.7 | 11.9 | 16.0 | 16.0 | 22.2 | 0.55  (0.31-0.99) | | 0.042 |
| ≥50mm/h | 2.9 | 3.7 | 4.6 | 5.9 | 7.6 | 7.6 | 7.6 |  |  |  |
| **IPS** | | | | | | | | | | |
| <3(ref cat) | 3.3 | 5.8 | 8.6 | 9.2 | 12.1 | 12.1 | 15.8 | 1.08  (0.57-2.04) | | 0.815 |
| ≥3 | 3.8 | 6.8 | 6.8 | 15.1 | 15.1 | 15.1 | 15.1 |  |  |  |
| **VLR risk score** | | | | | | | | | | |
| 0 factors (ref cat) | 0.6 | 0.6 | 2.4 | 2.4 | 2.4 | 2.4 | 2.4 |  | | |
| 1 factor | 2.8 | 3.6 | 5.3 | 6.4 | 11.0 | 11.0 | 11.0 | 4.31  (1-18.56) | | 0.050 |
| 2 factors | 4.9 | 9.1 | 11.6 | 11.6 | 17.2 | 17.2 | 17.2 | 7.84  (1.82-33.67) | | 0.006 |
| 3-4 factors | 11.0 | 14.2 | 17.5 | 27.3 | 27.3 | 27.3 | 39.3 | 15.35  (3.54-66.58) | | <0.001 |

*Pts=Patients, sHR = sub Hazard Ratio, 95% CI = 95% Confidence Intervals, ref cat = reference category, cHL= classical Hodgkin Lymphoma, NS=Nodular Sclerosis, MC=Mixed Cellularity, NLPHL=Nodular Lymphocyte-Predominant Hodgkin Lymphoma, ESR= Erythrocyte Sedimentation Rate, IPS= International Prognostic Score, VLR= Very Late Relapses, VLR risk score = No of risk 4 factors (MC histology, ESR <50mm/h, no RT, age ≥45 years)*

**Fine and Gray model*

# **Supplementary Table 3**

Rates of very late relapse at different time-points and univariate prognostic factor competing risks regression analysis for patients treated with ABVD or equivalent regimens (n=1042).

| **Overall outcomes** | **10-year** | **15-year** | **20-year** | **25-year** | **30-year** | **35-year** | **40-year** | **Overall** | |  |
| --- | --- | --- | --- | --- | --- | --- | --- | --- | --- | --- |
| Pts remaining at-risk  at each time point | 649 | 376 | 199 | 97 | 25 | 25 | 25 | 1042 | |  |
| Cumulative number of VLRs until each time point | 28 | 39 | 45 | 48 | 50 | 50 | 50 | 50 | |  |
| Cumulative incidence  (95% CI) | 3.1%  (2.1-4.4) | 5.2%  (3.7-7.0) | 7.2%  (5.1-9.7) | 8.9%  (6.2-12.3) | 12.7%  (7.2-19.8) | 12.7%  (7.2-19.8) | 12.7%  (7.2-19.8) | 12.7%  (7.2-19.8) | |  |
|  | | | | | | | | | | |
| **Cumulative incidence according to patients’ characteristics (%)** | | | | | | | | | **sHR (95% CI)** | ***p*- value*** |
| **Age** | | | | | | | | | | |
| <45 years (ref cat) | 2.2 | 3.6 | 6.1 | 7.2 | 12.0 | 12 | 12 | 2.14  (1.20-3.81) | | 0.01 |
| ≥45 years | 6.2 | 11.0 | 11.0 | 15.4 | 15.4 | 15.4 | 15.4 |  |  |  |
| **Gender** | | | | | | | | | | |
| Male | 4.0 | 5.8 | 8.3 | 10.7 | 14.8 | 14.8 | 14.8 | 1.44  (0.81-2.54) | | 0.211 |
| Female (ref cat) | 2.1 | 4.5 | 5.9 | 7.0 | 9.2 | 9.2 | 9.2 |  |  |  |
| **Stage** | | | | | | | | | | |
| IΑ/IIA (ref cat) | 2.4 | 3.8 | 7.2 | 8.9 | 22 | 22 | 22 | 1.30  (0.74-2.30) | | 0.364 |
| ΙΒ/IIB/III/IV | 4.1 | 7.3 | 7.3 | 8.9 | 10.8 | 10.8 | 10.8 |  |  |  |
| **B-symptoms** | | | | | | | | | | |
| No (ref cat) | 2.6 | 4.1 | 7.1 | 8.6 | 17.4 | 17.4 | 17.4 | 1.35  (0.76-2.42) | | 0.306 |
| Yes | 4.3 | 7.7 | 7.7 | 9.6 | 11.6 | 11.6 | 11.6 |  |  |  |
| **Histology** | | | | | | | | | | |
| MC | 5.2 | 10.8 | 13.2 | 17.3 | 23.2 | 23.2 | 23.2 | 2.89  (1.63-5.15) | | <0.001 |
| All other cHL(ref cat) | 2.3 | 3.1 | 5.2 | 6.0 | 8.0 | 8.0 | 8.0 |  |  |  |
|  | | | | | | | | | | |
| NLPHL | 5.6 | 9.0 | 9.0 | 9.0 | 9.0 | 9.0 | 9.0 | 2.01  (0.67-6.05) | | 0.213 |
| Non MCcHL(ref cat) | 2.3 | 3.1 | 5.2 | 6.0 | 8.0 | 8.0 | 8.0 |  |  |  |
| **Radiotherapy** | | | | | | | | | | |
| No (ref cat) | 5.4 | 9.4 | 10.5 | 13.1 | 16.1 | 16.1 | 16.1 | 0.51  (0.29-0.89) | | 0.019 |
| Yes | 2.3 | 3.9 | 6.1 | 7.4 | 12.2 | 12.2 | 12.2 |  |  |  |
| **Anemia** | | | | | | | | | | |
| No (ref cat) | 3.1 | 4.1 | 6.8 | 9.8 | 16.1 | 16.1 | 16.1 | 1.05  (0.59-1.86) | | 0.870 |
| Yes | 3.2 | 7.4 | 8.1 | 8.1 | 8.1 | 8.1 | 8.1 |  |  |  |
| **Leukocyte count** | | | | | | | | | | |
| <10x10^9^/L (ref cat) | 4.3 | 6.0 | 8.4 | 10.1 | 10.1 | 10.1 | 10.1 | 0.69  (0.39-1.24) | | 0.214 |
| ≥10x10^9^/L | 1.6 | 4.3 | 5.9 | 7.8 | 17.4 | 17.4 | 17.4 |  |  |  |
| **Lymphopenia** | | | | | | | | | | |
| No (ref cat) | 3.3 | 5.6 | 7.7 | 8.3 | 12.9 | 12.9 | 12.9 | 0.59  (0.14-2.41) | | 0.462 |
| Yes | 2.4 | 2.4 | 2.4 | 23.7 | 23.7 | 23.7 | 23.7 |  |  |  |
| **Albumin** | | | | | | | | | | |
| ≥4 g/dL (ref cat) | 3.3 | 5.0 | 6.9 | 8.3 | 8.3 | 8.3 | 8.3 | 1.19  (0.65-2.19) | | 0.574 |
| <4 g/dL | 3.3 | 6.2 | 7.1 | 9.5 | 12.7 | 12.7 | 12.7 |  |  |  |
| **ESR** | | | | | | | | | | |
| <50mm/h (ref cat) | 4.2 | 6.2 | 9.0 | 12.2 | 18.1 | 18.1 | 18.1 | 0.53  (0.27-1.04) | | 0.067 |
| ≥50mm/h | 2.3 | 3.3 | 4.4 | 4.4 | 7.3 | 7.3 | 7.3 |  |  |  |
| **IPS** | | | | | | | | | | |
| <3 (ref cat) | 3.1 | 5.3 | 7.6 | 8.4 | 14.7 | 14.7 | 14.7 | 1.23  (0.63-2.42) | | 0.544 |
| ≥3 | 4.1 | 7.6 | 7.6 | 14 | 14 | 14 | 14 |  |  |  |
| **VLR risk score** | | | | | | | | | | |
| 0 (ref cat) | 0.7 | 0.7 | 3.1 | 3.1 | 3.1 | 3.1 | 3.1 |  | | |
| 1 | 2.2 | 2.6 | 4.7 | 6.2 | 10.0 | 10.0 | 10.0 | 2.94 (0.67-13) | | 0.155 |
| 2 | 4.9 | 9.1 | 12.0 | 12.0 | 21.2 | 21.2 | 21.2 | 6.56  (1.52-28.35) | | 0.012 |
| 3-4 | 10.9 | 15.2 | 15.2 | 24.1 | 24.1 | 24.1 | 24.1 | 11.47  (2.6-51.64) | | 0.001 |

*Pts=Patients, sHR = sub Hazard Ratio, 95% CI = 95% Confidence Intervals, ref cat = reference category, cHL= classical Hodgkin Lymphoma, NS=Nodular Sclerosis, MC=Mixed Cellularity, NLPHL=Nodular Lymphocyte-Predominant Hodgkin Lymphoma, ESR= Erythrocyte Sedimentation Rate, IPS= International Prognostic Score, VLR= Very Late Relapses, VLR risk score = No of risk 4 factors (MC histology, ESR <50mm/h, no RT, age ≥45 years)*

**Fine and Gray model*

# **Supplementary Table 4**

Rates of very late relapse at different time-points and univariate prognostic factor competing risks regression analysis for patients with cHL treated with ABVD or equivalent regimens (n=978).

| **Overall outcomes** | **10-year** | **15-year** | **20-year** | **25-year** | **30-year** | **35-year** | **40-year** | **Overall** | |  |
| --- | --- | --- | --- | --- | --- | --- | --- | --- | --- | --- |
| Pts remaining at-risk  at each time point | 606 | 346 | 182 | 86 | 23 | 7 | NA | 978 | |  |
| Cumulative number of VLRs until each time point | 25 | 35 | 41 | 44 | 46 | 46 | 46 | 46 | |  |
| Cumulative incidence  (95% CI) | 3.0%  (2-4.3%) | 5.0%  (3.5-6.9%) | 7.2%  (5.0-9.8%) | 9.2%  (6.2-12.8%) | 13.6%  (7.4-21.7%) | 13.6%  (7.4-21.7%) | 13.6%  (7.4-21.7%) | 13.6%  (7.4-21.7%) | |  |
|  | | | | | | | | | | |
| **Cumulative incidence according to patients’ characteristics (%)** | | | | | | | | | **sHR (95% CI)** | ***p*- value*** |
| **Age** | | | | | | | | | | |
| <45 years (ref cat) | 2.0 | 3.2 | 5.9 | 7.1 | 12.7 | 12.7 | 12.7 | 2.51  (1.39-4.53) | | 0.002 |
| ≥45 years | 6.7 | 11.9 | 11.9 | 16.6 | 16.6 | 16.6 | 16.6 |  |  |  |
| **Gender** | | | | | | | | | | |
| Male | 4.4 | 6.0 | 8.9 | 11.7 | 16.6 | 16.6 | 16.6 | 1.81  (0.99-3.31) | | 0.053 |
| Female (ref cat) | 1.5 | 4.0 | 5.5 | 6.6 | 9.0 | 9.0 | 9.0 |  |  |  |
| **Stage** | | | | | | | | | | |
| IΑ/IIA (ref cat) | 2.4 | 4.0 | 7.7 | 9.5 | 22.6 | 22.6 | 22.6 | 1.15  (0.64-2.09) | | 0.634 |
| ΙΒ/IIB/III/IV | 3.8 | 6.6 | 6.6 | 8.6 | 10.7 | 10.7 | 10.7 |  |  |  |
| **B-symptoms** | | | | | | | | | | |
| No (ref cat) | 2.5 | 4.1 | 7.4 | 9.0 | 17.9 | 17.9 | 17.9 | 1.29  (0.70-2.36) | | 0.411 |
| Yes | 4.1 | 7.1 | 7.1 | 9.4 | 11.8 | 11.8 | 11.8 |  |  |  |
| **Histology** | | | | | | | | | | |
| MC | 5.2 | 10.8 | 13.2 | 17.3 | 23.2 | 23.2 | 23.2 | 2.87  (1.61-5.12) | | <0.001 |
| All other cHL(ref cat) | 2.3 | 3.1 | 5.2 | 6.0 | 8.0 | 8.0 | 8.0 |  |  |  |
| **Radiotherapy** | | | | | | | | | | |
| No (ref cat) | 5.8 | 9.2 | 10.4 | 13.5 | 17.2 | 17.2 | 17.2 | 0.47  (0.26-0.84) | | 0.011 |
| Yes | 2.1 | 3.7 | 6.2 | 7.5 | 12.7 | 12.7 | 12.7 |  |  |  |
| **Anemia** |  | | | | | | | | | |
| No (ref cat) | 3.1 | 4.1 | 7.3 | 10.8 | 19.1 | 19.1 | 19.1 | 0.93  (0.51-1.70) | | 0.811 |
| Yes | 3.0 | 6.7 | 7.5 | 7.5 | 7.5 | 7.5 | 7.5 |  |  |  |
| **Leukocyte count** | | | | | | | | | | |
| <10x10^9^/L (ref cat) | 4.5 | 6.4 | 9.1 | 11 | 11 | 11 | 11 | 0.55  (0.30-1.03) | | 0.061 |
| ≥10x10^9^/L | 1.1 | 3.5 | 5.2 | 7.3 | 17.9 | 17.9 | 17.9 |  |  |  |
| **Lymphopenia** | | | | | | | | | | |
| No (ref cat) | 3.1 | 5.4 | 7.6 | 8.3 | 13.7 | 13.7 | 13.7 | 0.64  (0.16-2.60) | | 0.529 |
| Yes | 2.5 | 2.5 | 2.5 | 23.7 | 23.7 | 23.7 | 23.7 |  |  |  |
| **Albumin** | | | | | | | | | | |
| ≥4 g/dL (ref cat) | 3.4 | 4.8 | 6.9 | 8.4 | 8.4 | 8.4 | 8.4 | 1.11  (0.58-2.11) | | 0.749 |
| <4 g/dL | 2.9 | 5.9 | 6.8 | 9.4 | 13.1 | 13.1 | 13.1 |  |  |  |
| **ESR** | | | | | | | | | | |
| <50mm/h (ref cat) | 4.1 | 6.4 | 9.4 | 13.0 | 19.6 | 19.6 | 19.6 | 0.54  (0.27-1.06) | | 0.071 |
| ≥50mm/h | 2.4 | 3.3 | 4.6 | 4.6 | 7.6 | 7.6 | 7.6 |  |  |  |
| **IPS** | | | | | | | | | | |
| <3 (ref cat) | 2.9 | 5.0 | 7.5 | 8.4 | 16.3 | 16.3 | 16.3 | 1.31  (0.66-2.59) | | 0.445 |
| ≥3 | 4.2 | 7.7 | 7.7 | 14.1 | 14.1 | 14.1 | 14.1 |  |  |  |
| **VLR risk score** |  | | | | | | | | | |
| 0 (ref cat) | 0.7 | 0.7 | 3.2 | 3.2 | 3.2 | 3.2 | 3.2 |  | | |
| 1 | 1.8 | 2.3 | 4.6 | 6.2 | 10.8 | 10.8 | 10.8 | 2.68 (0.59-12.05) | | 0.200 |
| 2 | 5.1 | 9.4 | 12.3 | 12.3 | 21.6 | 21.6 | 21.6 | 6.75  (1.56-29.23) | | 0.011 |
| 3-4 | 11.0 | 15.3 | 15.3 | 24.2 | 24.2 | 24.2 | 24.2 | 11.53  (2.56-52) | | 0.001 |

*Pts=Patients, sHR = sub Hazard Ratio, 95% CI = 95% Confidence Intervals, ref cat = reference category, cHL= classical Hodgkin Lymphoma, NS=Nodular Sclerosis, MC=Mixed Cellularity, NLPHL=Nodular Lymphocyte-Predominant Hodgkin Lymphoma, ESR= Erythrocyte Sedimentation Rate, IPS= International Prognostic Score, VLR= Very Late Relapses, VLR risk score = No of risk 4 factors (MC histology, ESR <50mm/h, no RT, age ≥45 years)*

**Fine and Gray model*

# **Supplementary Figure 1**

Study flow-chart (CT/CMT= Chemotherapy/Combined Modality Treatment, RT= Radiotherapy, CR1= 1st complete remission).


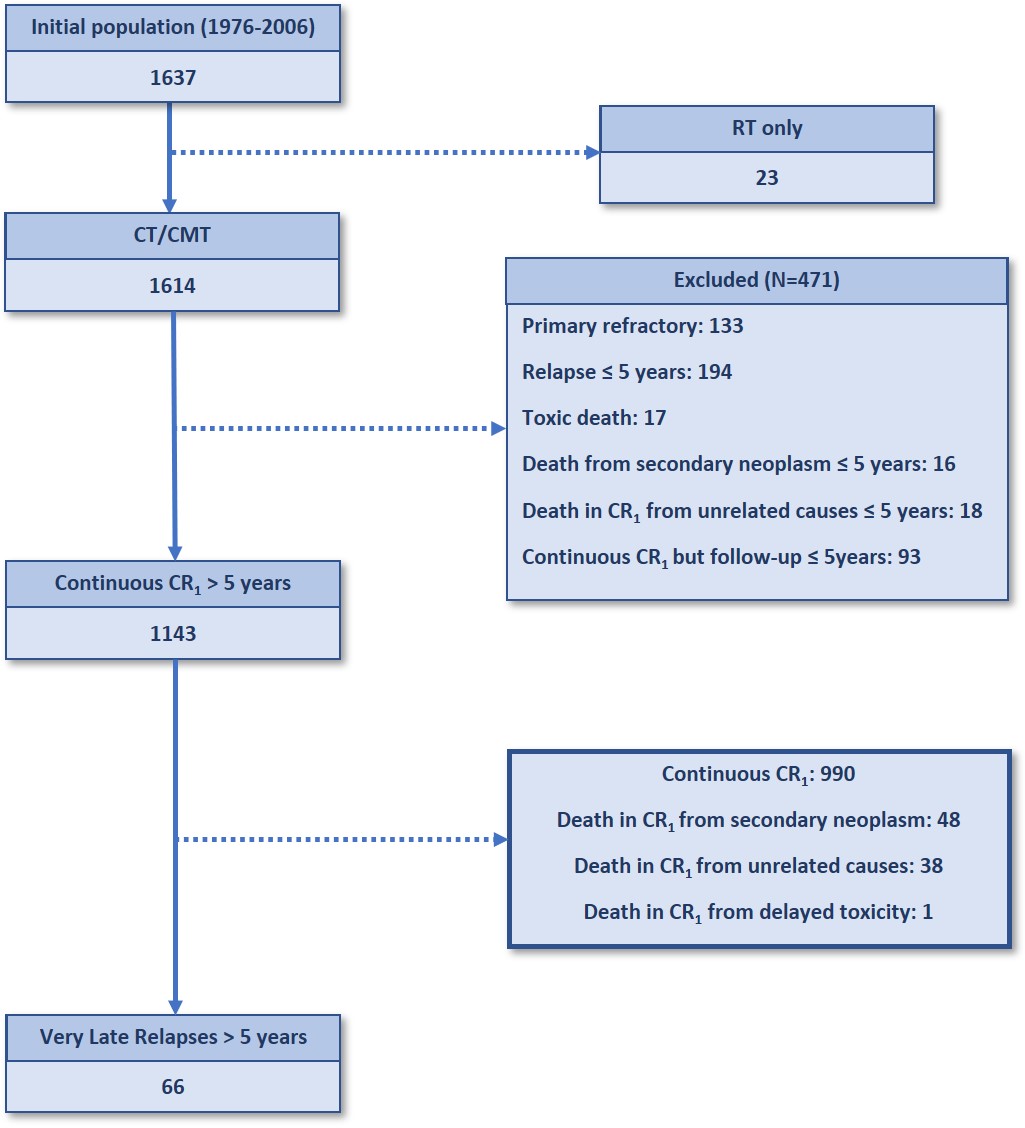


# **Supplementary Figure 2**

Cumulative Incidence of Very Late Relapses and competing-risks-adjusted Cumulative Incidence at different time-points from diagnosis in the whole patient population and according to chemotherapy regimen.


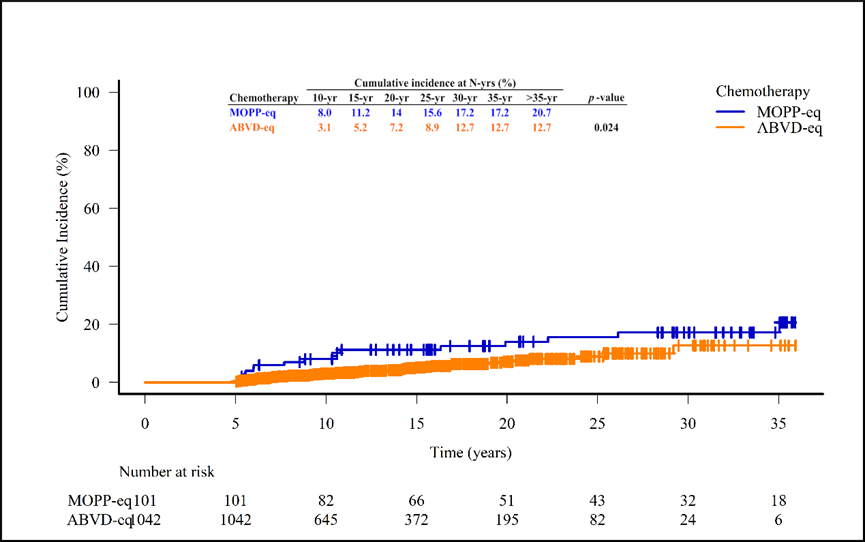


# **Supplementary Figure 3**

Predicted Cumulative Incidence curves of Very Late Relapses according to risk groups as derived from the competing risk multivariate model (VLR= Very Late Relapses) for the whole patient population (a), patients treated with ABVD or equivalent regimens (b), and patients with classical Hodgkin Lymphoma treated with ABVD or equivalent regimens (c).

C-index was 0.683 (95% CI 0.537-0.738) for the total cohort; 0.692 (95% CI 0.622-0.854) for the ABVD-treated cohort; and 0.713 (95% CI 0.524-0.861) for the cHL ABVD-treated cohort, suggesting good discriminative power.

#
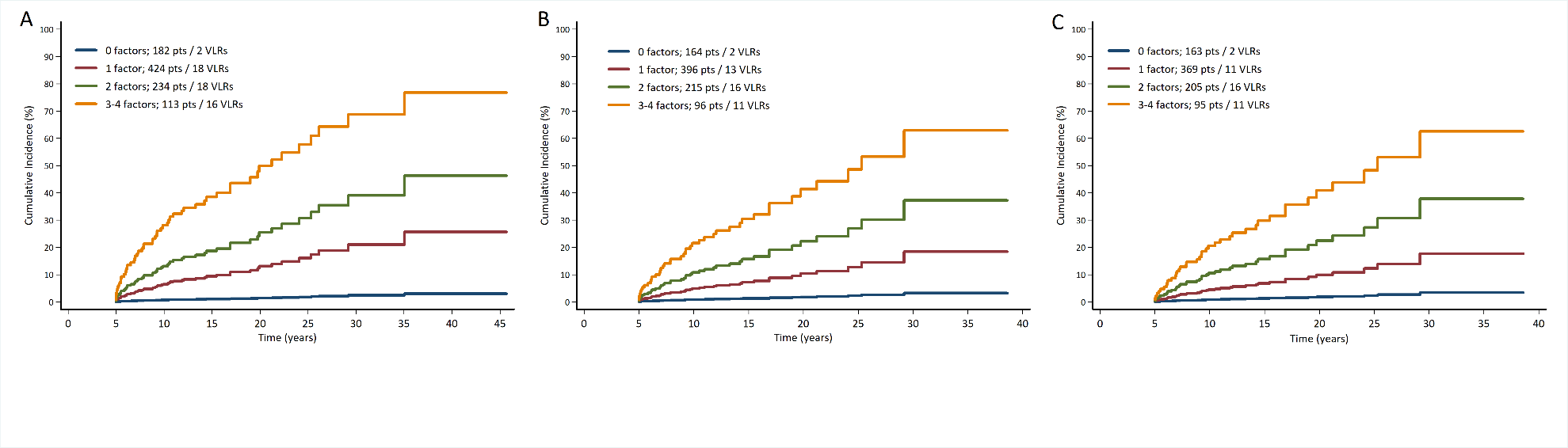


# **References**

1. Vassilakopoulos TP, Angelopoulou MK, Siakantaris MP, Kontopidou FN, Dimopoulou MN, Kokoris SI, et al. Combination chemotherapy plus low-dose involved-field radiotherapy for early clinical stage Hodgkin's lymphoma. Int J Radiat Oncol Biol Phys 2004; 59(3): 765-81.

2. Vassilakopoulos TP, Angelopoulou MK, Siakantaris MP, Kontopidou FN, Dimopoulou MN, Barbounis A, et al. Prognostic factors in advanced stage Hodgkin's lymphoma: the significance of the number of involved anatomic sites. Eur J Haematol 2001; 67(5-6): 279-88.

3. Vassilakopoulos TP, Nadali G, Angelopoulou MK, Dimopoulou MN, Siakantaris MP, Kontopidou FN, et al. beta(2)-microglobulin in Hodgkin's lymphoma: prognostic significance in patients treated with ABVD or equivalent regimens. J BUON 2005; 10(1): 59-69.

4. Vassilakopoulos TP, Dimopoulou MN, Angelopoulou MK, Petevi K, Pangalis GA, Moschogiannis M, et al. Prognostic Implication of the Absolute Lymphocyte to Absolute Monocyte Count Ratio in Patients With Classical Hodgkin Lymphoma Treated With Doxorubicin, Bleomycin, Vinblastine, and Dacarbazine or Equivalent Regimens. Oncologist 2016; 21(3): 343-53.

5. Karakatsanis S, Panitsas F, Arapaki M, Galopoulos D, Asimakopoulos JV, Liaskas A, et al. Serum Ferritin Levels in Previously Untreated Classical Hodgkin Lymphoma: Correlations and Prognostic Significance. Leuk Lymphoma 2021; (in press).

6. DeVita VT, Jr., Simon RM, Hubbard SM, Young RC, Berard CW, Moxley JH, 3rd, et al. Curability of advanced Hodgkin's disease with chemotherapy. Long-term follow-up of MOPP-treated patients at the National Cancer Institute. Ann Intern Med 1980; 92(5): 587-95.

7. Angelopoulou MK, Vassilakopoulos TP, Siakantaris MP, Kontopidou FN, Boussiotis VA, Papavassiliou C, et al. EBVD combination chemotherapy plus low dose involved field radiation is a highly effective treatment modality for early stage Hodgkin's disease. Leuk Lymphoma 2000; 37(1-2): 131-43.

8. Bonadonna G, Valagussa P, Santoro A. Alternating non-cross-resistant combination chemotherapy or MOPP in stage IV Hodgkin's disease. A report of 8-year results. Ann Intern Med 1986; 104(6): 739-46.

9. Klimo P, Connors JM. MOPP/ABV hybrid program: combination chemotherapy based on early introduction of seven effective drugs for advanced Hodgkin's disease. J Clin Oncol 1985; 3(9): 1174-82.

10.Vassilakopoulos TP, Rontogianni P, Angelopoulou MK, Assimakopoulos I, Boutsikas G, Chatziioannou S, et al. Early Interim Positron Emission Tomography (PET) Evaluation of Response after 2 ABVD Cycles in Advanced Hodgkin Lymphoma (HL): 8-Year Experience in Hellenic Departments – Conclusions and Limitations. 23rd Congress of EHA, June 14-17, Stockholm, Sweden, HemaSphere Abstract Book 2018; 2 (Suppl1)(s1): 535.

11. Schemper M, Smith TL. A note on quantifying follow-up in studies of failure time. Control Clin Trials 1996; 17(4): 343-6.

12. Gray RJ. A Class of K-Sample Tests for Comparing the Cumulative Incidence of a Competing Risk. Ann Stat 1988; 16(3): 1141-54.

13. Fine JP, Gray RJ. A Proportional Hazards Model for the Subdistribution of a Competing Risk. J Am Stat Assoc 1999; 94(446): 496-509.

14. Harrell FE, Jr., Lee KL, Mark DB. Multivariable prognostic models: issues in developing models, evaluating assumptions and adequacy, and measuring and reducing errors. Stat Med 1996; 15(4): 361-87.

15. Newson RB. Comparing the predictive powers of survival models using Harrell's C or Somers' D. SJ 2010; 10(3): 339-58.

16. Shoenfeld D. Chi-squared goodness-of-fit tests for the proportional hazards regression model, , Volume 67, Issue 1, 1980, Pages. Biometrika 1980; 67(1): 145–53.

17. Kanda Y. Investigation of the freely available easy-to-use software 'EZR' for medical statistics. Bone Marrow Transplant 2013; 48(3): 452-8.
